# Emerging ethical perspectives in the clustered regularly interspaced short palindromic repeats genome-editing debate

**DOI:** 10.2217/pme-2016-0047

**Published:** 2016-10-28

**Authors:** Silvia Camporesi, Giulia Cavaliere

**Affiliations:** 1 Director, Bioethics & Society Postgraduate Programme, Department of Global Health & Social Medicine, King's College London, London, UK; 2 Wellcome Trust PhD Student in Society & Ethics, Department of Global Health & Social Medicine, King's College London, London, UK

**Keywords:** bioethics, CRISPR, embryo research, gene editing, germline, stem cells

## Abstract

This paper provides an overview of the ethical issues in the international clustered regularly interspaced short palindromic repeats (CRISPR) genome editing debate from March 2015 to September 2016. We present the regulatory framework for embryo research in the UK, and explain why CRISPR is not a significant break with the past. We discuss the ethical issues arising from CRISPR applications beyond human embryos, namely the use of gene drive-engineered mosquitoes to eradicate diseases, engineering nonhuman animals to harvest organs for human transplant and engineering crops. We discuss the experiments that have demonstrated the technical feasibility of cultivating embryos *in vitro* for up to 14 days, and possibly beyond this limit, and the ethical issues arising from the proposal to extend the limit beyond 14 days.

First applied in mammalian cells in 2013, the CRISPR/Cas9-targeted genome-editing tool is an RNA-guided tool that consists of naturally occurring clustered regularly interspaced short palindromic repeats (CRISPR) and CRISPR-associated (Cas) nucleases (enzymes), which can generate DNA double-strand breaks at high efficiency to disrupt genes and insert desired DNA sequences. CRISPR/Cas9 is not the only existing genome-editing technique, but has advantages over others for its efficiency (lower number of off-target effects), versatility (the system works in all eukaryotic organisms) and accuracy (as it enables targeted editing). The total cost of the technique is as little as US$30, in what has been referred to as ‘democratization’ of the gene-editing technology [1]. The system is now ubiquitously used in biology laboratories worldwide for a variety of purposes and routine genetic modifications [2,3]. In this paper we will be referring to CRISPR technology as different nucleases can be associated with the repeats, although Cas9 is the one – thus far – predominantly used.

In the ‘CRISPR applications on human embryos: big ado about nothing?’ section, we introduce the applications on human embryos and the debate that has ensued internationally. In section ‘CRISPR & the regulation of embryo research in the UK: not a significant break with the past’ we describe the regulatory framework for research on human embryos in the UK and explain how CRISPR technology is positioned within this legal and ethical context. In the section ‘Which CRISPR futures? CRISPR applications beyond the human embryo’ we outline areas of ethical concern of CRISPR applications beyond human embryos, namely engineering mosquitoes to eradicate diseases; engineering nonhuman animals for human organ transplant; and engineering crops for human consumption. In the section ‘CRISPR embryo debate: momentum building outside the USA?’ we present some recent international developments of the debate on CRISPR applications on human embryos, and discuss the recent experiments demonstrating the feasibility to cultivate embryos *in vitro* for longer than the current limit of 14 days. In the last section of the paper we reflect on some possible ‘CRISPR futures’ and we conclude reiterating the importance of considering the non-human applications of the technology.

## CRISPR applications on human embryos: big ado about nothing?

The beginning of the CRISPR debate can be traced back to March/April 2015 and to the publication in the journal, *Protein & Cell*, by a group of Chinese scientists led by Huang of the first findings resulting from the application of CRISPR in nonviable (tripronuclear zygotes) human embryos [4]. Aware of the forthcoming publication by Chinese scientists, a group of US scientists led by Jennifer Doudna at UC Berkeley (one of the co-discoverers of CRISPR mechanisms, with Emmanuelle Charpentier at Humboldt University in Germany in the same team, and in the other team Fen Zhang at The Broad Institute, MIT) published a letter on 3 April 2015 to the journal, *Science*, recommending a ‘prudent pathway’ for genomic engineering and germline gene modification. The letter called for a self-imposed temporary moratorium on germline applications of CRISPR-Cas9 until further deliberation [5]. This call was reminiscent of the self-imposed temporary moratorium on recombinant DNA technologies of the Asilomar conference in 1975 (indeed, the parallels were explicit as Nobel prize winners, Paul Berg and David Baltimore, were two of the authors and the organizers of the Asilomar conference) [6]. D Baltimore and colleagues were not the only ones calling for prudency, other scientists from the Alliance for Regenerative Medicine in Washington, DC, USA led by Edward Lanphier (chairman of the Alliance) wrote a letter striking similar chords to the journal *Nature*, which is aptly titled, ‘Don't edit the human germline’ [7].

At the end of April 2015, the Director of the NIH, Dr Francis Collins, released a statement affirming that the NIH would not fund any use of gene-editing technologies on human embryos. The motivation was that “the concept of altering the human germline in embryos for clinical purposes has been debated over many years from many different perspectives, and has been viewed almost universally as a line that ‘should not be crossed’” [8]. Eric Lander, Director of the Broad Institute at MIT (to note – Fen Zhang, one of the inventors of the technology, is based at the Broad Institute at the MIT and working with George Church). echoed in the *New England Journal of Medicine* (under the not-particularly original heading of, ‘Brave New Genome’ of a need for ‘caution’ against ‘reckless’ applications and quotes a supposed ‘long-standing consensus’) that the human germline should not be crossed [9]. A similar stance was taken by the UNESCO International Bioethics Committee (IBC) in October 2015 [10]. The IBC is a permanent UNESCO body composed of 36 independent experts in the life sciences and humanities. It was established in 1993 and it is a global forum for in-depth reflection in bioethics. The Committee produces advisory documents and recommendations on specific bioethical issues which are then disseminated and transmitted to the Member States and the UNESCO Executive Board, but have no binding power on Member States.

However, it is not the case that editing the human germline is seen universally as a line that should not be crossed, as Collins, Lander and the IBC seem to purport. As a matter of fact, reactions to the application on human embryos of CRISPR varied significantly. In the UK, for example, where research on human embryos is allowed up to 14 days within the remit of the Human Embryology Act (see CRISPR & the regulation of embryo research in the UK: not a significant break with the past), British scientist, Lovell Badge stepped in to pronounce a statement against the moratorium:

“*I disagree with such a moratorium, which is in any case unlikely to be effective. I am fully supportive of research being carried out on early human embryos *in vitro*, especially on embryos that are not required for reproduction and would otherwise be discarded. If the techniques work, there are many interesting questions that could be asked about the role of specific genes in early human embryo development*” [11].

Voices from the British bioethics community also lined up to support the experiments. In June 2015, Oxford bioethicists Julian Savulescu, Jonathan Pugh, Thomas Douglas and Christopher Gyngell were the first to clearly position themselves in favor of CRISPR applications to embryo research. They published a perspective piece in the journal, *Protein & Cell* (to note, the same journal where the Chinese team published their article), where they argued that research applying CRISPR technology to the human embryos:

“*Far from being wrong … is a ‘moral imperative’ and that, “gene editing could significantly lower this disease burden thereby benefiting billions of people around the world over time. To intentionally refrain from engaging in life-saving research is to be morally responsible for the foreseeable, avoidable deaths of those who could have benefitted. Research into gene-editing is not an option, it is a moral necessity*” [12].

Similarly, prominent British philosopher John Harris argued that risks to alter future generations (and, possibly, to harm them) are not specific to CRISPR technology but are intrinsic in any new reproductive technology (including, natural reproduction), and that the ethical challenges raised by the use of CRISPR on human embryos had been overemphasized [13,14]. Harris concluded by flipping the argument on its head and arguing that safety issues should be understood as an argument in favor of doing further research, not of on banning it. Harris is also a member of the The Hinxton Group, an international research group created to address ethical and social issues concerning stem cells research and embryo research regulations. In a 2015 statement, the steering committee of The Hinxton Group defended the importance of going forward with basic research on human embryos (i.e., *in vitro*), while putting on hold clinical applications of CRISPR on human embryos [15].

Both Harris and Savulescu adopt a utilitarian approach to bioethics, according to which the criterion of right action is the principle of utility, and an action is morally right if it maximizes utility. Failing to continue research that could possibly be lifesaving is, from a utilitarian perspective, equal to killing people. According to the utilitarian standpoint, acts and omissions are morally equivalent, hence there is no morally relevant difference between killing and letting die [16]. Following this line of argument, it becomes evident why both so strenuously defend going forward with research using CRISPR on human embryos.

Precautionary positions toward CRISPR are based on two elements: the age-old fear of creating ‘designer babies’ or of the return of past eugenics; and the risk of unforeseen effects on future generations. These two worries express rather different underlying concerns, whereas the former is driven by the so-called ‘slippery slope argument’ that sounds familiar to philosophers and bioethicists alike [17], the latter is an instance of precautionary considerations driven by limits of knowledge and technical feasibility. According to the slippery slope argument, allowing a practice X although not particularly ethically troubling it itself (in this instance, allowing the use of gene-editing tools on human embryos *in vitro*) would initiate a process leading to unethical practices W, Y, Z (for instance, germline modifications of embryos for clinical applications). Albeit widely criticized in the philosophical arena [18–20], slippery slope arguments are very often used in debates on reproductive technologies and in policy making.

## CRISPR & the regulation of embryo research in the UK: not a significant break with the past

In September 2015, Dr Kathy Niakan, group leader at Francis Crick Institute (interestingly to note – the Francis Crick is a new molecular biology research center funded by six UK research institutions, which was not yet operational at the time of the application, and will be inaugurated in November 2016) in London, put forward the first application to the Human Fertilisation & Embryology Authority (HFEA) to carry out research using CRISPR on human embryos [21]. In the UK, CRISPR research on human embryos falls within the remit of the 1990 Human Embryology & Fertility Act, which allows research *in vitro* up to 14 days. Clinical applications – in other words, transfer to uterus of the embryos, are prohibited by law [22]. The HFEA oversees assisted reproduction (*in vitro* fertilization [IVF] and related practices such as preimplantation genetic diagnosis, as all clinics performing assisted reproduction services need to have been granted a license from the HFEA) and research on human embryos, which are supernumerary from IVF [22]. For research on human embryos to be permitted, a specific HFEA license needs to be granted outlining the details of the research project [22].

Positioning CRISPR research on human embryos within the context of the UK regulatory framework for embryo research is important to understand the social and political context that has fostered the support to CRISPR research in the UK. The 14-day limit for conducting research on human embryos came about as a result of the IVF Inquiry, which was established in 1982 following the birth of the first ‘test tube’ baby (IVF baby), Louise Brown, in 1978. Reflecting on this limit had become an urgent matter to address as IVF necessarily entails the creation of supernumerary embryos – that is, embryos that are not implanted *in utero* and whose further use (if any) needed to be decided. The committee, led by philosopher Mary Warnock, was established in 1982 with the task of developing a time limit for research on human embryos [23]. Mary Warnock's understanding of her role as chair of the committee was to reach an acceptable compromise between opposing views, a compromised based on a deliberative process. In the case of regulating research on human embryos, these incompatible moral premises would see at one end of the spectrum those who assumed a ‘sanctity of life’ position, and at the other end those who adopted a more utilitarian oriented perspective. Developmental biologist Anne McLaren was called in to provide her expertise to the committee in search for a compromise between the two opposed positions [23]. Anne McLaren recommended to limit research on human embryos to the 14th day of development as this marks the emergence of the primitive streak in the human embryo, which in turn signals the beginning of gastrulation – that is the first differentiation of the embryonic inner cell mass into three layers – endoderm, mesoderm and ectoderm – a third of which will later develop into the nervous system. Gastrulation also corresponds to the last point in embryonic development in which the embryo could cleave to twins. Hence the emergence of the primitive streak was framed as the beginning of individual development and the term ‘pre-embryo’ was coined to describe the pre 14-day embryo [23]. The Warnock report recommendation was enshrined into law in 1990, in what became the Human Fertilisation and Embryology Act, which led to the establishment of the HFEA.

In February 2016, the HFEA granted approval to Kathy Niakan's request [24]. The minutes of the decision (which are fully available here [25]) clearly indicate that the research project delineated by Niakan did not involve any clinical applications of the gene-edited embryos or gene-edited-derived human embryonic stem cells, nor involved researching for a time period longer than 14 days, thus falling squarely within the limit of the British law. The approval is conditional to the approval of the Institute's research ethics committee (see above) [25].

It will now be evident how in the UK CRISPR genome-editing technology applied on the human embryos represents only the latest type of technology to do research on embryos *in vitro*, rather than a significant break with the past. This is why the two main arguments outlined above against the use of CRISPR on human embryos were almost absent from the UK debate, with the exception of those who adopt a sanctity of life position and are against *in toto* to the use of human embryos for research [26].

As a matter of fact, Julian Hitchcock, a British lawyer specialized in the regulation of emerging life science technologies, commented that:

“*CRISPR-Cas9 is of obvious utility to any of the ‘principal purposes’ listed by the Human Fertilisation & Embryology Act, so the decision of the Human Fertilisation & Embryology Authority to permit it should never have been in doubt: Niakan might as well have applied to use a new sort of test tube*” [27].

## CRISPR applications beyond the human embryo

In this section we outline applications of CRISPR gene-editing technologies that have been to some extent overshadowed by the main focus of debate on CRISPPR applications to human embryos (with some notable early exceptions [28–31]). They involve respectively the use of CRISPR to eradicate disease vectors; to create humanized animal models for organ transplant; and to genetically engineer crops.

### CRISPR to eradicate disease vectors

Endonuclease genes such as Cas9 (or other Cas-related proteins) cut the corresponding chromosomal locus lacking them. This in turn induces the cell to repair the break by copying the nuclease (enzyme) gene onto the damaged chromosome via homologous recombination [32]. This mechanism, called ‘gene drive’ was developed in *Drosophila melanogaster* (i.e., the common fruit fly used ubiquitously as an animal model in molecular genetics laboratories worldwide) but is now being applied to other species such as *Aedes aegypti*, the carrier for dengue fever as well as Zika virus, to engineer the mosquitoes so that they produce only male offspring (that do not bite) [33]. When an organism carrying an engineered endonuclease gene drive mates with a wild-type organism, the gene drive is preferentially inherited by all offspring. This can enable the drive to spread until it is present in all members of the population. If the gene in question is propagated across multiple generations, the mosquitoes would eventually go extinct (pending acquiring of resistance) [32,33]. Drive-mediated genome alterations are not permanent on an evolutionary timescale, and would not be effective in species that reproduce asexually, with slow reproductive cycles or with closed reproductive niches. Hastings Center bioethicist, Gregory Kaebnick notes that “evolutionary processes would hardly be nullified by gene drives”, as the acquisition of resistance to gene drives is not a matter of ‘if’, but of ‘when’. However, he adds that, “Still, the basic point is rather shocking from an environmentalist's perspective: gene drives hold out the prospect of altering species in a way we have not been able to do before” [34].

Genetically engineered mosquitoes of the *A. aegypti* species have been developed by Oxitec – a British company purchased by the US synthetic biology company, Intrexon – and have already been released in the Piracicaba district in Brazil in 2015, with results indicating a reduction in wild mosquito larvae of 82% by the end of the year [35]. On 18 January 2016, the Brazilian city of Piracicaba has announced that it will expand the use of genetically modified mosquitoes to fight *A. aegypti* and the spread of Zika. In May 2016, the same company, Oxitec, announced that CRISPR-engineered mosquitoes would be released to the Cayman Islands to combat the spread of dengue, Zika and chikungunya (another type of tropical fever) [36]. The US FDA is currently considering an investigational trial for Oxitec solutions in the Florida keys, where cases of Zika have been recorded since July 2016, which if approved, would be the first application of CRISPR-engineered mosquitoes in the USA [37].

To control the unpredictable consequences on the ecosystem, scientists have proposed that a reverse engineering system could be built in the engineered species as a way of providing ‘intrinsic safeguards’ if the original drive system fails to perform as desired. Methods of confining gene-drive systems to local populations are also being explored including ‘daisy gene-drive systems’, which may be ‘powerful enough to eliminate all copies of an unwanted global drive system via local immunizing reversal or population suppression before disappearing themselves’ [38]. Applications to the control of invasive species through gene drive have also been envisaged, as gene-drive applications to improve the sustainability and safety of pesticides and herbicides [33].

While the aforementioned applications are all examples of ‘well-meant’ applications of gene drive, it is not implausible to imagine that the same technology could also be used for the opposite purposes of releasing in the environment disease-carriers engineered mosquitoes. Hence, CRISPR in this context represents a perfect instance of a dual-use dilemma of research whose ethical implications demand to be considered [39,40].

The ‘disruption of natural order’ is an example of a classic bioconservative worry. According to this view, ‘tampering’ or ‘meddling’ with nature is intrinsically wrong regardless of the consequences [41,42]. ‘Nature’ is assumed to hold an intrinsic moral value as the product of an intelligent design. We think, in line with others [43,44], that the ‘natural’ should not hold an intrinsic moral value. However, we think that the bioconservatives’ concerns should not be too quickly dismissed. The value of the argument lies not, in our opinion, on the sacrality of the natural order, but on another consideration: a genetic trait that may be deleterious in one context (e.g., thalassemia) and for the individual may turn out to be advantageous in another context (e.g., resistance to malaria). At the same time, what counts as normal is context dependent [45]. Along similar lines, Robin Lovell-Badge has pointed out, for example, that one of the applications of CRISPR in the context of embryo editing could be to delete the allele APOE4, which is the allele of the apolipoprotein E associated with Alzheimer's disease (with heterozygotes being approximately three-times and homozygotes 15-times more likely to develop the disease than homozygotes). However, Robin Lovell-Badge notes that, “with any risk allele, particularly a common one, it is important to ask why it is maintained in the population at a relatively high frequency, could APOE4 in fact confer some advantage to carriers unrelated to its connection to Alzheimer's?” [46]. There are very tangible risks – which are very difficult to predict – in deleting genetic diversity as we may end up deleting traits that can turn out advantageous in a different context, or advantageous for the species although, not for the individual.

### CRISPR applied to humanized animal models for organ transplant

The shortage of organs for human transplants is one of the longstanding unresolved medical problems worldwide. Ethical dilemmas arise when having to develop criteria for the allocation of a scarce resource. An increasing number of scholars are now portraying CRISPR technology as a way out of the shortage through the development of humanized animal models for organ transplant [47–50]. Human genes can be inserted into non-human animals (with pigs appearing as the most promising candidates) at the stage of blastocyst. Humanized animal models could then be developed from chimeric blastocysts, from which organs for human transplant could be harvested. Thanks to the human-inserted genes, such organs would not cause the problem of graft-versus-host disease, which leads to the transplanted organ being rejected by the host (and hence to organ failure and possible death of the host). This is not science fiction, although Margaret Atwood, in her book, *Oryx and Crake*, had anticipated this scenario in 2003. In early August 2016, the NIH announced plans to lift the moratorium – which was put in place in September 2015, before any funding of the kind had actually been issued – on human–animal chimera research and engaged in a heightened review of the research [51,52]. According to Inso Hyun, Associate Professor of Bioethics & Philosophy at Case Western University, who has written an op-ed in *PLoS Biology*, “It is easy to overstate the concern about the moral humanization of acute human/nonhuman chimeras” [50].

Hyun's type of reasoning falls along the well-established lines of beneficence-based arguments, which are the main arguments in support of carrying out this type of research. Indeed, writes Hyun: “Given the noble aims of this research, it is puzzling to some why the NIH is so nervous about providing federal funds to researchers with a track record of success in this area” (page 1) and adds figures to point out the current shortage of human organs for transplantation in the USA, and what this research would do to solve this. In general, beneficence-based arguments are structured as follows: if humanized animal models for organ transplant can be the solution to the longstanding problem of shortage of organs, then we have strong beneficence reasons to go forward. The argument from beneficence and the moral imperative to relieve suffering are often used to support scientific advances in the face of possible public resistance and disagreement. However, we believe that concerns about human–animal chimeras should not be dismissed too quickly, in a similar way to concerns about releasing gene-drive engineered mosquitoes in the ecosystem. The value of the concerns lies, once again, not in a supposed sacrality of human nature, which would be supposedly corroded by the animal genes – in this, we agree with Hyun – but in an open discussion about resource allocation and prioritization untouched by Hyun. To put it simply, we should seriously consider if and why we cannot address the problem of shortage of human organs in another way which does not lead to human–animal chimeras, for example, through changes in our opt-in policies to donate organs and facilitate ways to make that possible. We should be wary of arguments that appeal uncritically to an ‘apparent and urgent’ and ‘the humanitarian importance’ of research (page 2). Nothing is apparent or urgent; we always need to carefully consider alternatives, and in the case of organs for human transplants, there are alternatives.

In addition, cross-species experiments raise other longstanding discussions concerning the moral status of non-human animals and their use in research for the benefit for our species [53–55], and the derivation of chimeric and hybrid human embryonic stem cells [56–58]. These are not new ethical issues but need to be addressed *de novo* given the technical feasibility allowed by CRISPR of growing humanized organs in nonhuman animals.

### CRISPR applications in agriculture: does it still count as genetically modified organisms?

CRISPR technologies have far-reaching applications in agriculture [59]. While transgenic crops have existed for years, there are important regulatory questions that need to be answered, and social and ethical issues around genetically modified organisms for food consumption ‘crop up’ again (pun intended). In April 2016, the company, DuPont Pioneer, announced plans to market CRISPR-modified corn, soybeans, canola, rice and wheat. The engineered crops will have drought resistance and higher yields [60]. The US Department of Agriculture shortly thereafter announced that it does not consider the CRISPR corn “as regulated by USDA Biotechnology Regulatory Services”. Why is that so? As noted by Caplan *et al*., “What makes CRISPR different from other methods of agricultural genetic engineering is that it no longer requires the insertion of foreign DNA into the plant genome using a virus, bacterial plasmid or other vector system” [40]. In other words, CRISPR-edited organisms would no longer classify as transgenic organisms in *sensu stricto* as there is no insertion of foreign DNA [61]. The same issue is being debated in Europe too, where historically there has been a higher opposition to transgenic crops. In Sweden, authorities recently said that CRISPR-edited plants should not be defined a genetically modified organisms (GMO) under EU legislation.

Introducing a new powerful technique in a contested and arguably lagging legal framework might prove to be irresponsible and further endanger public trust in expert knowledge in a context where this is already low. The European Commission was expected to give guidance on what products of genome editing would be classed as GMOs by the end of 2015, but in March 2016 an EC spokesperson commented that the “outcome and timeline cannot be pre-empted for the time being”. This creates a climate of uncertainty that, according to Huw Jones, member of the GMO panel European Food Safety Authority, and many others in the business who share his view, stifles innovation [62].

The report on genetically engineered crops published in May 2016 by the US National Academy of Sciences advisory group provides an example of the unsettled ethical issues at stake. According to the report, there is no ‘substantiated’ evidence that genetically engineered crops might be dangerous for human health and damaging for the environment. In spite of this, the controversy about GMOs remains as it reaches beyond health concerns to issues of public trust in expert knowledge. The assumption often made that a knowledge deficit from the part of the public (the so-called ‘public deficit model’) [63] underlies the public resistance to GMO has been dismantled by many science and technology scholars over the past 20 years [64–68]. This needs to be acknowledged by policy makers if progress in this area is to be made. Public trust in expert knowledge is possibly at historical lows in the western world (with resistance to vaccination, climate change, evolution, just to name a few). Social scientists and bioethicists need to work together to seriously consider the underlying causes of resistance to science, which cannot be fixed by a presumption that the public needs more information. A responsible approach to the use of CRISPR for GMOs and gene drive needs to bring together biology and ecology. As put by American science and technology scholar, Emma Frow, “it is not about the ethical issues of the technology that we need to decide, it is about which ‘collective futures’ we want to shape for our society, and planet, with CRISPR” [69].

## CRISPR embryo debate: momentum building outside the USA?

The international controversy on CRISPR research on human embryos is far from being settled. In April 2016, a second paper by Chinese scientists, led by Fan, using CRISPR in human embryos was published in the *Journal for Assisted Reproduction and Genetics* [70]. To note, this is a different research group from the first one who published in *Protein and Cell* in 2015: the former researchers are based at Sun Yat-sen University, whereas the latter are based at Guangzhou Medical University. The authors successfully introduced a naturally occurring allele, which is aimed at conferring HIV resistance, into early human tripronuclear (nonviable) zygotes by CRISPR-mediated genome editing. The modified embryos displayed mosaicism in which wild-type cells and genetically modified cells coexisted. It should be noted that tripronuclear zygotes were the same choice of biological model as the one made by the group led by Huang *et al*. in 2015 [4]. It can be argued that the choice of using tripronuclear zygotes by both Chinese research groups was an explicit strategy to pre-empt criticism by the international community for working on human embryos.

Meanwhile, other researchers in Europe have announced they plan to apply CRISPR technology on human embryos. One example is Frederick Lanner, group leader at Karolinska Institute in Sweden, who in an interview with the journal *Nature* in April 2016 [71] discussed his plans – approved by his home institution – to genetically engineer human embryos in order to understand the biology of preimplantation development building on his previous work [72], which is a goal not dissimilar from Niakan's research project. A government bioethics panel in Japan also recently gave the green light to basic research *in vitro* on human embryos, but not to clinical applications, mirroring the HFEA regulation of embryo research in the UK [73].

It seems therefore that (at the time of finalizing this article, September 2016) there is a building momentum on research on human embryos outside the USA. This momentum is echoed by recent experiments conducted by two teams of scientists, one based at Rockefeller University in the USA and the other at the Wellcome Trust Stem Cell Institute and The Gurdon Institute in Cambridge in the UK. These experiments, published in two separate articles – *Nature* and *Nature Cell Biology* – in May 2016 demonstrated for the first time that embryos can be cultured *in vitro* for 12–14 days [74,75]. Prior to these findings, scientists were able to culture embryos *in vitro* until the 9th day. Early reactions from both scientists and bioethicists to these experiments have been favorable and enthusiastic both in the UK and in the USA, with some exceptions from scholars who are outright against using embryos for research [29,76]. British bioethicist, John Harris, already quoted above, has taken a clear position in favor of redrawing the 14-day rule. According to Harris, whereas previously there was no need or reason to revise (i.e., extend) the limit, the fact that embryos can now be cultured *in vitro* beyond the 14th day makes the case for a change in the law, on the basis of the expected benefits that this research could bring to humanity [77]. Along similar lines, the director of germline and epigenomic research at The Gurdon Institute in Cambridge, UK, Azim Surani, maintained before these recent findings emerged that there was a strong case for extending the time frame for research on human embryos beyond 14 days in light of the expected benefits of research and the scarce knowledge of early embryo development in humans [78].

In contrast to the regulation of embryo research in the UK, in the USA there is no equivalent of a central regulatory body such as the HFEA. In the USA, the Director of the NIH, and the President of the USA have the discretion to prohibit federal funding to research that they do not approve (as was formerly the case with President Bush and research on human embryonic stem cells) [79]. This does not mean that private funds cannot be invested in research, although the burden of justification and persuading investors, is on the privately funded laboratories. For example, in California the Institute for Regenerative Medicine announced that they will finance research on CRISPR on human embryos [80]. Dr Eli Adashi, former Dean of Medicine and Biological Sciences at Brown University, recently argued that cultural differences play a role in the formulation of science policies, and that “the divergent outlooks of the United Kingdom and the United States on human embryo research [are] informed by dissimilar positions across the prochoice/prolife divide. These distinctions are very much in evidence in the regulatory arena” [79]. While the UK seems to be characterized by a ‘compromise-seeking’ attitude toward bioethical controversies (evident in Mary Warnock's IVF Inquiry), the USA seems to be much more polarized at the two extremes. Isasi and Knoppers’ results of a survey of policy approaches to embryonic stem cell research in 50 countries position the UK at the most liberal end of the spectrum [81]. However, the position of the UK is less permissive than it seems, as the Human Embryology Act does not grant an umbrella permission to research on human embryos within the 14-day limit, but a specific license needs to be granted by HFEA to each research group following an application, and the license is a conditional approval which needs to be followed by the approval by a research ethics committee.

It should be made absolutely clear that a revision of the 14-day limit for research on human embryos in the UK would entail a parliamentary vote and HFEA approval to change the Human Fertilisation & Embryology Act. As explained in a section “CRISPR and the regulation of embryo research in the UK: not a significant break with the past”, the 14-day limit was put in place on the basis of biological and philosophical considerations, with the aim to find a workable solution in the face of moral disagreement on the status of the human embryo, and not due to technical limits alone. If scientists are now able to grow the embryos *in vitro* for longer than 14 days, it does not necessarily entail that they ought to do it. This line of reasoning is precisely what 17th century philosopher, David Hume condemned as an ‘inconceivable’ deduction of what one ought to do from a set of *is*-premises – in other words, from what one can do. In other words, Hume argued that no ethical conclusion, for example, “we ought to extend the 14-day limit for embryo research”, can be inferred from purely factual premises, such as, “scientists are able to grow embryos *in vitro* for >14 days”. A change in the law cannot rest solely on technical feasibility grounds. In addition, the potential benefits of extending the limit beyond 14 days should not be uncritically accepted on the basis of scientists’ assumptions (for a critique in relation to CRISPR-editing technology, see Jasanoff *et al.*, 2016) [82].

## Conclusion

In this paper we have provided an overview of the international CRISPR bioethics debate as it has unfolded since the publication in April 2015 of the first research paper by Huang *et al*. [4] of the applications of CRISPR technology in human embryos. As we are bioethicists working in the UK, we have contextualized the discussion on the application to human embryos of CRISPR within the British regulatory system. We have commented on the support that CRISPR research on human embryos has received in the UK, and argued that this support needs to be understood within the British sociopolitical context of embryo research since the establishment of the HFEA in 1990, where it does not represent a discontinuity with the past. We have also discussed the recent experiments that have demonstrated the technical feasibility of cultivating embryos *in vitro* for up to 14 days, and possibly beyond this limit. We do not believe that technical feasibility alone should drive a change in the law. In the central part of this paper we have pointed to some areas that deserve the attention of the bioethical community beyond human embryos, namely engineering mosquitoes to eradicate diseases, engineering animals to harvest organs for human transplant and engineering crops. To conclude, a critical analysis of pros and cons, which aims to avoid standardized patterns of reactions to the emergence of new technologies, is necessary to move forward in the CRISPR debate and extend it beyond the narrow focus on editing the human embryos. An interdisciplinary, international task-force that brings together scientists, bioethicists, social scientists, lawyers, policy makers and lay citizens, and that does not construe the ethical and the social issues of CRISPR technologies as ‘lagging behind’ the science, should be convened. It is important to remember that editing the human embryo with CRISPR technology is not the only way in which we can change future generations, and certainly not the way that would have the greatest impact on our planet.

## Future perspective

In spite of optimistic and often hyped previsions and of the importance to push forward scientific innovation, it is important to bear in mind that we are dealing with uncertainties, and should not take for granted the expected benefits of the CRISPR technology. We have seen similar arguments unleashed before for nanotechnology, synthetic biology, stem cell research and gene therapy. We agree with American historian of science Ben Hurlbut, who writes, “technological controversies have come and gone, but modes of reacting to them have come to be patterned and institutionalized” [83], while British sociologist Adam Hedgecoe has referred to this pattern as the “reinforcement of sociotechnical expectations” operated by bioethicists [84].

The debate on CRISPR is no exception in this respect. However, we should resist this standard mode of bioethics reacting to a new technology, and of policies ‘lagging behind science’, and be wary of accepting a linear model of innovation that presupposes that a new technology will bring about great benefits for society. A number of issues remain beyond our knowledge, the first of which is the complex relationship between genes and the environment. Intervening in human evolution might prove advantageous in a particular context at present, but not in another context in the future. In this sense, CRISPR can teach us some humility. It is the task of bioethicists, and social scientists to critically unpack the impacts, benefits, consequences, promises and fears of CRISPR technology.

Editing embryos (and the germ-line) in the context of basic research will likely reveal meaningful information concerning embryo development, possibly shedding light on the reasons behind early miscarriages and infertility, as argued by Niakan and Lanner. However, we agree with Lundberg and Novak [85], among others, that most of the clinical applications of CRISPR will not lie in editing the germline, but in editing somatic tissues. It may very well be that CRISPR represents the final upheaval of a gene therapy field that has had many ups and downs over the past 30 years. Indeed, the first gene therapy clinical trial in humans has been given the go ahead in China, and a similar one is expected to be given go ahead soon in the USA [86]. This clinical trial is designed for cancer patients in order to improve the success rate of gene therapies and to reduce the incidence of relapses. Other gene therapy trials may be approved in the near future. CRISPR applications to the development of humanized animal models for organ transplant, and the applications of CRISPR to eradicate disease vectors and other species with gene drive, are in our opinion two major breakthroughs of the technology that represent a discontinuity with the past. They deserve an ethical assessment in terms of allocation and prioritization of resources, including in terms of beneficence-based arguments and risk assessment.

In the context of assisted reproduction, gene editing will be used as an alternative preimplantation genetic diagnosis (PGD) in IVF for those rare cases in which parents homozygous for a lethal mutation (e.g., Huntington disease) want to have biologically related children and for which PGD is not an option (as all embryos are affected). If implemented in assisted reproduction, selected genes of embryos created through IVF will be modified with CRISPR *in vitro* prior to being transferred *in utero*. In this context, the question to ask is whether the quest for a biologically related child is warranted in the context of allocation of scarce resources, and with the existence of alternatives such as PGD that provide a viable alternative in most cases. Our society needs to discuss the meaning of biological kinship in light of the available alternatives, such as adoption. We need to ask ourselves: should we not invest more resources in making adoption possible? The same resource allocation questions should be asked in the context of creating chimeric animal models for organ donation: should we not invest more in facilitating ethical human organ donation?

**Executive summary**

This article explores the ethical issues raised by the application to human embryos of CRISPR Cas/9 from March 2015 to April 2016.
It shows the necessity of widening the attention beyond the focus on human embryo to other nonhuman applications.

**Clustered regularly interspaced short palindromic repeats research on human embryos**

Clustered regularly interspaced short palindromic repeats (CRISPR) is a ubiquitously used technology in laboratories worldwide thanks to its versatility as it is applicable to all eukaryotic cells.
First applied in mammalian cells in 2015, CRISPR became the center of a global debate in April 2015 due to the announcement of a group of Chinese scientists that they had applied the technology on human nonviable embryos.
Negative reactions to the Chinese announcement came largely from the USA, featured appeals to the Precautionary Principle and to slippery slope arguments, and led to a self-imposed moratorium on CRISPR research on human embryos.
In the UK, many scientists and ethicists lined up in favor of allowing basic research with CRISPR on human embryos.

**Positioning CRISPR research within the regulation of embryo research in the UK**

In the UK, embryo research is allowed up until the 14th day of development; thus, allowing research with CRISPR technology on embryos does not represent a significant breach with the past.
Reactions from the UK can be explained if we position the application of CRISPR to human embryos within the regulation on embryo research (Human Embryology Act, 1990).

**CRISPR applications beyond the human embryo**

There are a number of current and possible future applications of CRISPR gene-editing technologies that do not involve the human embryo and deserve ethical attention. These include:
  Gene drive to genetically engineered mosquitoes to eradicate disease vectors. Ethical issues: effects on ecosystem, altering biodiversity, dual use of research.
  CRISPR to create humanized animal models for human organ transplant. Ethical issues: cross-species experiments, allocation and prioritization of resources toward creation of chimeric animal models for organ transplants instead of implementing policies to facilitate organ donation from humans to humans.
  CRISPR to genetically engineer crops. Ethical issues: Uncertainty in terms of regulation as genetically modified crops may not count as GMOs.

**CRISPR research on human embryos: momentum building outside the USA?**

The debate on CRISPR has not been settled: in this section we present some of the most recent developments involving the use of CRISPR on human somatic cells and the present state of the debate on embryo research. Other countries such as Sweden and Japan are going forward with CRISPR applications on human embryos.

**Future perspective**

Therapeutic potential of CRISPR lies in applications to somatic cells (gene therapy) rather than in germline cells.
In the context of assisted reproduction preimplantation genetic diagnosis is a viable alternative to CRISPR except in rare cases of dominant genetic conditions, where CRISPR could represent a viable alternative for parents who are carriers of the dominant genetic condition and want to have biologically related children.
